# Cardiac Surgery: A Matter of Life or Death

**DOI:** 10.5812/traumamon.14880

**Published:** 2013-10-13

**Authors:** Hamidreza Taghipour

**Affiliations:** 1 Department of Cardiothoracic Surgery, Trauma Research Center, Baqiyatallah University of Medical Sciences, Tehran, IR Iran

**Keywords:** Cardiac Surgical Procedures, Suicidal Ideation, Professional Competence

**Figure 1.**
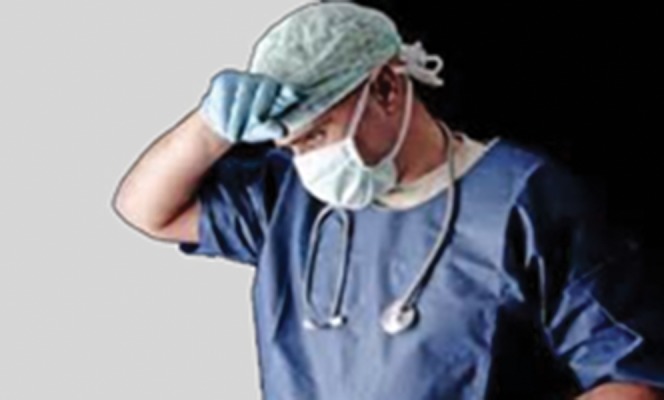
The “Stressed-Out” Surgeon Showing Signs of Postoperative Fatigue

Cardiovascular surgery, also known heart surgery is a stressful profession where surgical skills and experience can make the difference between life and death for the cardiac patient. The cardiac surgeon must acquire numerous years of education, training and surgical expertise. Open heart surgical operations are lengthy taking 4 - 6 hours or longer to complete. The surgeon has to be on call all the time because emergencies can occur anywhere, anytime, preoperatively or postoperatively without warning. The cardiac surgeon has to work with a very experienced team; even a small mistake may lead to a tragic unexpected cascade of events, resulting in a disaster. Thus, stress is an integral part of cardiovascular surgery. It is not surprising that surgeons practicing this stressful profession aged 45 and older have suicidal rates 1.5 to 3 times that of the general population (1). In a study in 2011 which assessed 8000 surgeons, 501 reported suicidal ideation (2). These surgeons are most likely “stressed-out” (Figure 1). 

A competent cardiovascular surgeon should be able to battle stress, “keep his cool” under pressure, “think on his feet” and improvise “on-the-spot” in case something unexpectedly goes awry during the course of the surgery. Moreover, the task of a cardiovascular surgeon does not end in the operatory. It is imperative that he or she has good bedside manners in order to effectively communicate with heart patients and their guardians in the ward, discuss treatment options preoperatively as well as help the patients recover postoperatively. The surgeon should also be in good physical shape to be able to stand for hours without fatigue. Thus, regular exercise is also important. Mentality, psyche and self-confidence are equally vital to combat the inherent stress of open heart surgery. However, for the cardiac surgeon preferring a stress-less work environment, teaching may be a good albeit less lucrative alternative (3-5).
